# Residual errors in visuomotor adaptation persist despite extended motor preparation periods

**DOI:** 10.1152/jn.00301.2021

**Published:** 2022-01-19

**Authors:** Matthew Weightman, John-Stuart Brittain, R. Chris Miall, Ned Jenkinson

**Affiliations:** ^1^School of Sport, Exercise and Rehabilitation Sciences, University of Birmingham, Birmingham, United Kingdom; ^2^School of Psychology, University of Birmingham, Birmingham, United Kingdom; ^3^MRC-Versus Arthritis Centre for Musculoskeletal Ageing Research, University of Birmingham, Birmingham, United Kingdom; ^4^Centre for Human Brain Health, University of Birmingham, Birmingham, United Kingdom

**Keywords:** mental rotation, motor preparation, sensorimotor adaptation

## Abstract

A consistent finding in sensorimotor adaptation is a persistent undershoot of full compensation, such that performance asymptotes with residual errors greater than seen at baseline. This behavior has been attributed to limiting factors within the implicit adaptation system, which reaches a suboptimal equilibrium between trial-by-trial learning and forgetting. However, recent research has suggested that allowing longer motor planning periods prior to movement eliminates these residual errors. The additional planning time allows required cognitive processes to be completed before movement onset, thus increasing accuracy. Here, we looked to extend these findings by investigating the relationship between increased motor preparation time and the size of imposed visuomotor rotation (30°, 45°, or 60°), with regard to the final asymptotic level of adaptation. We found that restricting preparation time to 0.35 s impaired adaptation for moderate and larger rotations, resulting in larger residual errors compared to groups with additional preparation time. However, we found that even extended preparation time failed to eliminate persistent errors, regardless of magnitude of cursor rotation. Thus, the asymptote of adaptation was significantly less than the degree of imposed rotation, for all experimental groups. In addition, there was a positive relationship between asymptotic error and implicit retention. These data suggest that a prolonged motor preparation period is insufficient to reliably achieve complete adaptation, and therefore, our results suggest that factors beyond that of planning time contribute to asymptotic adaptation levels.

**NEW & NOTEWORTHY** Residual errors in sensorimotor adaptation are commonly attributed to an equilibrium between trial-by-trial learning and forgetting. Recent research suggested that allowing sufficient time for mental rotation eliminates these errors. In a number of experimental conditions, we show that although restricted motor preparation time does limit adaptation—consistent with mental rotation—extending preparation time fails to eliminate the residual errors in motor adaptation.

## Graphical abstract

**Figure:**
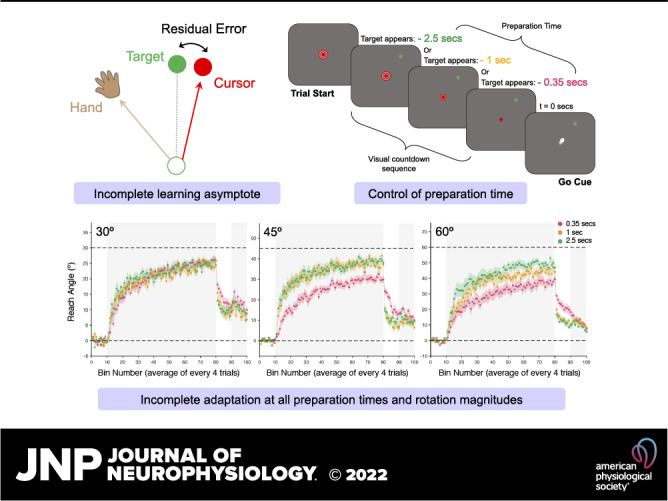

## INTRODUCTION

Sensorimotor adaptation has been extensively studied using visuomotor rotations (1–3). In this task, individuals adapt reaching movements to counter visual feedback that is rotated, for example, 30° or 45° from the hand’s position. However, adaptation is typically incomplete, and performance plateaus with errors a few degrees greater than at baseline, regardless of the rotation magnitude imposed (4–9). State-space models of learning accurately capture this incomplete compensation (10, 11). In these models, error-driven learning and forgetting (or a reversion to baseline) work in opposition, and equilibrate below optimal performance, resulting in the commonly observed persistent error.

However, Vaswani et al. (7) demonstrated that under certain conditions, individuals disengage from this limited error-dependent learning to attain greater task success. In one of their experimental conditions, Vaswani et al. (7) “clamped” visual feedback after an initial learning block, such that a small, fixed, visual error was presented, regardless of movement accuracy. They found that under these conditions, participants appeared to select an alternative, more exploratory learning policy, which enabled them to close the errors and even to overcompensate for the rotation (which differed from the partial reversion to baseline predicted by the state-space model). The authors suggested that the altered feedback distribution triggered alternative learning processes (and enabled the elimination of residual errors), which are usually suppressed during naturalistic feedback conditions.

More recently, Langsdorf et al. (9) presented an alternative theory to explain why under normal, nonclamped environments, the central nervous system fails to eliminate residual errors during motor adaptation. They suggested an intrinsic speed-accuracy trade-off: where time-consuming planning processes are interrupted by the imperative onset of movement, resulting in fast, but inaccurate, movements. In their study, they showed that when an obligatory 2.5 s wait period was introduced between target presentation and movement initiation, participants were able to fully adapt to a 45° rotation, leaving no persisting errors at the end of learning. However, if this wait period was not enforced or was introduced at the end of the movement, when no planning was assumed to be taking place, participants failed to fully counteract the rotation. The authors highlighted mental rotation as a time-consuming cognitive process potentially involved in the planning of visuomotor adaptation.

For some time now, visuomotor adaptation has been framed as a combination of distinct learning processes (12–16). These processes are customarily described as implicit and explicit. The implicit component adapts slowly and is driven by sensory prediction errors (10, 17). The explicit component is a fast adaptation process and involves, among other processes, strategic reaiming to counter the detected perturbation (15, 18, 19). Such strategies are thought to include a form of mental rotation (20). Langsdorf et al. (9) suggest that under naturalistic feedback conditions, parametric mental rotations from the target to the intended movement goal are prematurely terminated (despite no time constraints) and result in aimed movement trajectories falling short of the imposed rotation.

Behavioral and neurophysiological research suggests a role of mental rotation in the planning of movements aimed at angles away from visually defined targets (21–25). Neuronal population vectors recorded in the monkey motor cortex gradually rotate from a stimulus direction to a cued movement direction during the planning of a reach (23, 24). In addition, the completion of mental rotation tasks requires long reaction times with larger magnitudes of rotation (21, 26); a signature of cognitive strategies (4, 20, 27).

Given this knowledge, we aimed to extend the findings of Langsdorf et al. (9), to further understand the roles of extended planning periods and mental rotation in attaining full adaptation. We designed an online visuomotor adaptation task, where participants had either long (2.5 s), medium (1 s), or short (0.35 s) enforced preparation periods between target presentation and movement onset and were required to adapt to either a small (30°), moderate (45°), or large (60°) visuomotor rotation. Previous studies (9) suggest that the 45° rotation would be fully corrected with the longest preparation period, errors for the middle interval would be greater, and a substantial residual error should be found for the shortest preparation interval. This negative relationship between preparation interval and residual error should be most obvious for the 60° rotation; the smallest rotation (30°) might allow full compensation even at the shortest preparation interval. In other words, the response time could be considered the sum of a mental rotation period linearly related to the rotation magnitude and a fixed movement initiation period; if the sum exceeds the prescribed preparation interval, then residual errors should be seen.

## MATERIALS AND METHODS

### Participants

A total of 200 participants were recruited via posters, via study adverts, and through personal contact (age = 18–37 yr, means ± SD = 22.4 ± 3.5 yr, 89 males); all gave written informed consent before participating and either received no remuneration or received course credit that counted toward their university degree mark. The study was approved by the University of Birmingham Ethics Board (Science, Technology, Engineering and Mathematics Ethical Review Committee). Participants were self-reported as right-handed (*n* = 181) or left-handed (*n* = 19) and used their preferred hand to complete the task (all but 5 of the left-handed participants completed the task using their right hand). All participants had normal, or corrected to normal, vision and reported no history of neurological disease. Initially, 180 participants were pseudorandomized into one of nine experimental groups (each *n* = 20), which differed in the amount of preparation time provided (0.35, 1, or 2.5 s) and the magnitude of cursor rotation (30°, 45°, or 60°). These participants formed the online arm of the study (Table 1). To avoid any possible confounds relating to online data collection and only after COVID-19 restrictions relating to in-person human testing were lifted, we also collected data from an additional experimental group who completed the task in a laboratory setting. Participants in this group experienced a 45° cursor rotation, with 2.5 s of preparation time (Table 1)—a closer replication of the study by Langsdorf et al. (9).

**Table 1.** Summary of experimental groups

	0.35 s			1 s			2.5 s			
	30°	45°	60°	30°	45°	60°	30°	45°	45° (Lab)	60°
*n* (*n* male)	20 (9)	20 (6)	20 (8)	20 (11)	20 (12)	20 (4)	20 (10)	20 (13)	20 (7)	20 (9)
Mean age, yr (±SD)	23.5 (±2.0)	25.4 (±3.4)	25.9 (±3.4)	21.0 (±1.1)	21.5 (±3.7)	22.6 (±5.0)	20.8 (±1.3)	21.0 (±1.6)	20.2 (±1.8)	22.7(±3.7)

### Task

#### Online.

**Figure 1.**
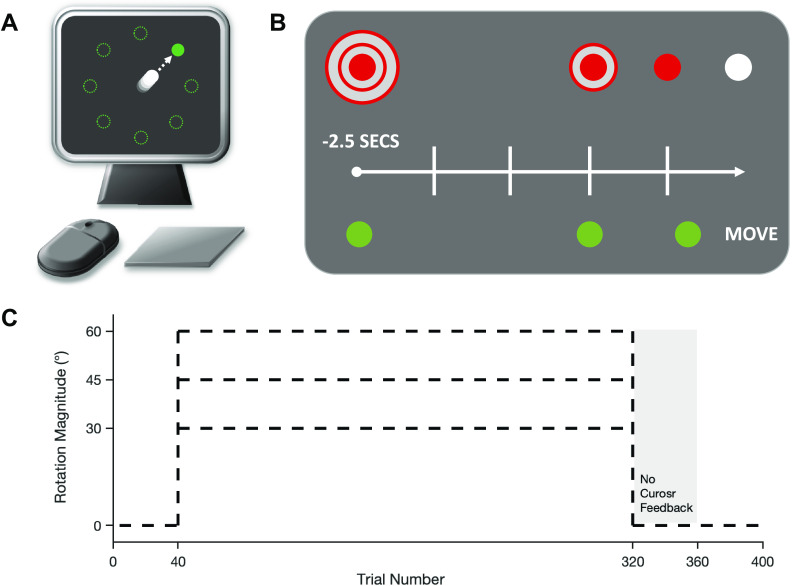
The experimental design. *A*: participants used either a mouse or trackpad to direct an on-screen cursor toward targets presented radially around a central starting position. *B*: an example of the cursor countdown sequence and movement cue. At the beginning of each trial, the (red) cursor would appear in the center of the screen, flanked by two larger (red) rings (shown here on the *top*). During the trial, the two rings would disappear in sequence and the cursor would change color to white. Participants were required to initiate their movement in synchrony with the cursor color change, using the disappearing rings as a countdown. The target (shown on the *bottom*) would appear either 2.5, 1, or 0.35 s before the movement cue, depending on group assignment. *C*: time course of the study protocol. Participants completed 40 baseline trials, followed by 280 adaptation trials (where either a 30°, 45°, or 60° cursor rotation was imposed), then 40 no-feedback trials, and finally 40 de-adaptation trials.

We developed a visuomotor adaptation task using the behavioral science experiment platform PsychoPy (28), which was implemented online via Pavlovia (https://pavlovia.org). Participants were required to have access to a desktop or laptop computer with internet connection and a mouse or trackpad to navigate through and complete the task (*n* = 117/180 participants used a trackpad; *n* = 63/180 participants used a mouse). Participants were sent a link to access the task and entered a unique code that determined which combination of preparation time and rotation angle they would encounter. Participants were asked to sit in a location of their choosing, so that they could easily see their computer screen and comfortably reach and manipulate the mouse or trackpad. They made center-out movements with either their mouse or on their trackpad from an on-screen central starting position to targets that appeared on an invisible circular array surrounding it (Fig. 1*A*). They were told to make one fast and straight “shooting” movement, aiming to get the on-screen cursor as close as possible to the target. Prior to starting the task, all participants completed a brief tutorial, which included the task instructions and aims. At this point, participants were asked to contact a researcher if they were unclear about any part of the task and/or had any questions relating to the instructions.

#### Laboratory.

Participants sat in an armless chair in front of a computer monitor (21.5-inch iMac, 60 Hz refresh rate) and used a trackpad (Apple Magic Trackpad) to control the on-screen cursor. The trackpad measured 0.49–1.09 cm in height, 16 cm in width, and 11.49 cm in depth and was fixed to the desk next to the computer screen. A 1-cm movement on the trackpad corresponded to ∼3 cm of on-screen cursor movement. Crucially, the participants received the same instructions and completed the same tutorial before starting the task. The remaining task parameters were exactly as the online paradigm. All 20 participants were right-handed (self-report) and used their right hand to complete the task.

#### Protocol.

At the beginning of every trial, the cursor (red filled circle) would be located in the center of the screen (gray background). The cursor was surrounded by two larger red annuli, which formed a bullseye-like formation (Fig. 1*B*). At a set moment, the outer ring disappeared, followed by the inner ring 0.5 s later, and the cursor then changed color from red to white after a further 0.5 s. Participants were told that this sequence of events should be treated as a countdown to movement and that they should aim to time their movement so that it was initiated in synchrony with the cursor changing color from red to white (Fig. 1*B*). Depending on the experimental group, the target (green filled circle) would appear either 2.5 s, 1 s, or 0.35 s before the movement cue, thus creating three distinct preparation periods. The target could appear at any one of eight possible locations, equally spaced (separated by 45°) around an invisible circular array centered on the start position, and pseudorandomized so that the target appeared at each location once every cycle of eight trials. The position at which the cursor passed the invisible perimeter was displayed for 0.5 s as a static open white circle, providing feedback of movement accuracy. After an intertrial interval of 0.5 s, the cursor would reset to the central position ready for the next trial. Participants were encouraged to move their hand back to a comfortable starting position after each trial. We did not acquire details regarding the different screen sizes used by each participant in the online experiment; therefore, we cannot stipulate the exact dimensions of the various stimuli. However, all elements of the visual display were scaled according to the size of the task window and thus were the same relative size for all participants. Consequently, the physical movement size required varied between participants depending on their setup and was not recorded beyond input device. For the in-laboratory experiment, the stimuli dimensions were as follows: cursor diameter = 1 cm, target diameter = 1.2 cm, and target circle radius = 10.8 cm. If participants attempted to move 0.15 s before or after the go-cue, or if movement time exceeded 0.25 s, the message “Response too fast/slow” appeared on the screen and the trial was aborted. These measures were to encourage fast movements, initiated correctly with the task instructions.

### Experimental Design

Participants first performed eight practice trials (one at each target location). These trials were performed with veridical cursor feedback and were used to familiarize the participants with the timing of their version of the task. The main task directly followed the practice trials and included four phases: baseline, adaptation, no-feedback, and de-adaptation (Fig. 1*C*). During baseline trials (*n* = 40), participants received veridical cursor feedback, such that the on-screen cursor moved in accordance with participants’ movements. Adaptation trials immediately followed, in which the cursor feedback was rotated 30°, 45°, or 60° clockwise (depending on the experimental group, see Table 1) relative to participants’ movement. This block lasted 280 trials, selected to ensure learning approached its asymptotic limit, based on pilot data collected before the present study. Although there is some indication that full asymptotic saturation of learning was not achieved in all conditions, regression analysis of the last 40 trials of adaptation indicated that there was no significant trend remaining (all *P* > 0.19, with all slopes between 0.041 and −0.039), and thus, we use the term “asymptote” to refer to the final state reached at the end of the 280 adaptation trials. Following adaptation trials, participants performed no-feedback trials (*n* = 40). During these trials, participants were told to stop using any strategies they might have used to achieve the task objectives and try to aim directly for the target. The cursor was hidden at all times during the trial, apart from when located in the central position for the countdown cue. End-point error was not displayed during the no-feedback phase. Veridical cursor feedback and display of end-point error were then restored for the final de-adaptation trials (*n* = 40).

There were 10 participant groups (Table 1), to cover all combinations of preparation time (long: 2.5 s, medium: 1 s, and short: 0.35 s) and the magnitude of cursor rotation (large: 60°, moderate: 45°, and small: 30°), with the 2.5 s/45° group repeated in a laboratory setting. The preparation time was held constant throughout the whole task; the rotation was applied only during the adaptation phase.

### Data Analysis

There were three main outcome variables: reach angle, response time, and movement duration. Our primary dependent variable—reach angle—was the angular difference between the target location and participants’ movement at end-point (i.e., the difference in angle between the vector linking the starting position and the target marker and the vector linking the starting position and the point at which participants’ movement crossed the target circle perimeter). Participants were excluded from analysis if they either failed to follow task instructions (i.e., ignoring the rotated cursor and not adjusting their aiming direction) or if they violated the timing limits of the task on four successive trials (indicating a lack of concentration or distraction). Seventeen individuals were excluded based on this criterion and were replaced with new participants to achieve the desired sample size. Trials were deemed outliers and removed if they fell 2.5 standard deviations outside the group average on each trial. A total of 1.23% of all trials were removed from further analysis. Data from each participant were then averaged into bins of four trials to be used for visual representation and statistical analysis. Response time was defined as the time period between target presentation and movement onset (i.e., cursor movement breaking an invisible line immediately surrounding the starting position), and movement duration was defined as the time period between movement onset and when movement crossed the target circle. Reach angle data was not recorded for trials where the response or movement time limits were violated.

### Statistical Analysis

Asymptotic levels of adaptation were defined as the mean error over the last 40 trials of the adaptation phase and compared between groups in a two-way ANOVA [Rotation Magnitude = 3 levels (30°/45°/60°) × Preparation Time = 3 levels (0.35 s/1 s/2.5 s)], with any significant main effects or interactions followed up with Bonferroni-corrected multiple comparisons. Asymptotic differences from the imposed rotation magnitude were compared using a Wilcoxon signed-rank test, after data from some groups failed normality checks (Shapiro–Wilk test). Mean reach angles across all baseline trials and during the first 16 no-feedback trials for each participant were used for baseline and retention comparisons. Data from the laboratory group were processed and analyzed separately from the online data, as this group was not factored into the original study design. In-laboratory data were compared with their online counterpart using unpaired *t* tests (two-tailed) and from the degree of imposed rotation using a Wilcoxon signed-rank test. All statistical analyses were carried out in MATLAB (R2018b) and SPSS (IBM, version 27). All ANOVAs were run as general linear models. The threshold for statistical significance was set at *P* < 0.05, and we report *W*, *F*, *T*, and *P* values, as well as effect sizes from ANOVAs [partial eta squared (${\text{η}}_{\text{p}}^{2}$)].

## RESULTS

### Baseline Performance Does Not Differ between Groups

**Figure 2.**
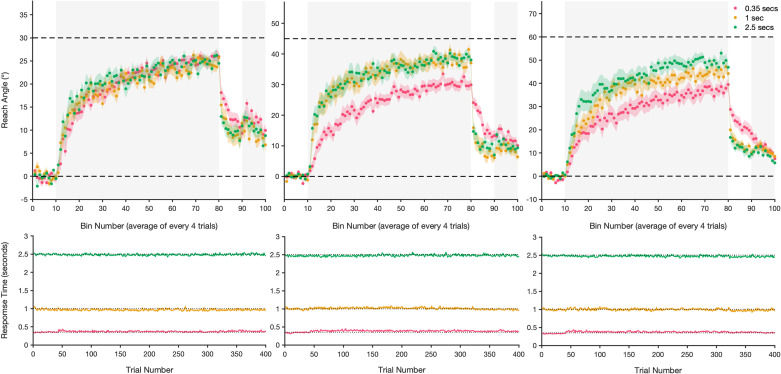
Mean reach angle and response time for each of the online experimental groups. *Top*: reach error (±standard error, shaded region) is averaged every four trials into bins for (from *left* to *right*) the 30°, 45°, and 60° groups, during baseline, adaptation (shaded gray background), no feedback, and de-adaptation trials (shaded gray background). Zero degrees and the magnitude of imposed rotation are shown by horizontal dashed lines. *Bottom*: mean response time (time between target presentation and movement initiation), ±standard error (shaded region) for each group. Note, preparation times were predetermined at 0.35, 1, or 2.5 s (dotted lines) and were tightly controlled.

Differences in reach angle during the baseline phase were assessed in a two-way ANOVA (Rotation Magnitude × Preparation Time), which revealed no significant main effects for rotation magnitude [*F*(2,171) = 0.071, *P* = 0.93, ${\text{η}}_{\text{p}}^{2}$ = 0.001] and preparation time [*F*(2,171) = 0.3, *P* = 0.75, ${\text{η}}_{\text{p}}^{2}$ = 0.003] and no significant Rotation Magnitude × Preparation Time interaction [*F*(4,171) = 0.45, *P* = 0.77, ${\text{η}}_{\text{p}}^{2}$ = 0.1]. This result suggests that all groups performed similarly during baseline trials (Fig. 2), and thus, group differences are unlikely to influence performance later in the task. We felt this analysis was important, given the limited experimenter input associated with online research.

### The Effect of Differing Preparation Time on Asymptotic Levels of Adaptation

**Figure 3.**
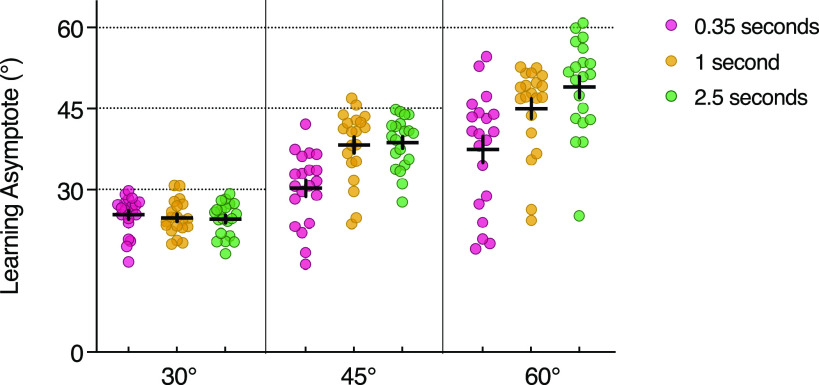
The asymptotic levels of adaptation for each participant in each of the rotation magnitude and preparation time groups (online experiment). Filled circles represent the adaptation asymptote for each participant (defined as the average of the last 40 adaptation trials), with mean values and standard error depicted by horizontal and vertical lines, respectively. Dashed horizontal lines depict the imposed cursor rotation for each group.

A two-way ANOVA comparing asymptotic levels of adaptation revealed significant main effects for rotation magnitude [*F*(2,171) = 121.01, *P* < 0.001, ${\text{η}}_{\text{p}}^{2}$ = 0.59] and preparation time [*F*(2,171) = 15.17, *P* < 0.001, ${\text{η}}_{\text{p}}^{2}$ = 0.15) and a significant Rotation Magnitude × Preparation Time interaction [*F*(4,171) = 5.24, *P* < 0.001, ${\text{η}}_{\text{p}}^{2}$ = 0.11]. Multiple comparisons revealed that in the 45° rotation group, there were some differences between learning asymptotes, with regard to preparation time provided. The 0.35-s preparation time group displayed impaired adaptation compared with the 1- and 2.5-s groups (both *P* < 0.001), with no further differences between the latter two conditions (*P* > 0.99). However, contrary to the study by Langsdorf et al. (9), all final levels of adaptation significantly differed from 45° [0.35 s: *W* = −210.0, *P* < 0.001, 95% confidence interval (CI) = (−16.72, −11.32); 1 s: *W* = −200.0, *P* < 0.001, 95% CI = (−9.71, 2.50); 2.5 s: *W* = −210.0, *P* < 0.001, 95% CI = (−9.33, 3.18)]. The 0.35-s preparation time group compensated 67.3% for the rotation, with the 1- and 2.5-s groups achieving 85.1% and 86.1% compensation, respectively (Figs. 2 and 3), suggesting that additional preparation time allowed for greater compensation but not the elimination of residual errors.

Similarly, for the 60° rotation condition, shortening planning time to 0.35 s significantly reduced the final level of adaptation compared with both 1 s and 2.5 s preparation periods (0.35 s vs. 1 s: *P* = 0.001, 0.35 s vs. 2.5 s: *P* < 0.001). However, there was no difference between the 1- and 2.5-s groups with respect to final adaptation levels (*P* = 0.17). Participants averaged 62.4% compensation with 0.35 s preparation time, 75.0% with 1 s preparation time, and 81.7% with 2.5 s preparation time (Figs. 2 and 3). In addition, all asymptotic levels of adaptation significantly differed from the imposed 60° rotation [0.35 s: *W* = −210, *P* < 0.001, 95% CI = (−31.14, −16.02); 1 s: *W* = −210.0, *P* < 0.001, 95% CI = (−16.27, −10.27); 2.5 s: *W* = −206.0, *P* < 0.001, 95% CI = (−16.76, −6.44)].

In the 30° rotation group, there were no differences in asymptotic error between any of the different preparation time groups (all *P* > 0.99, Fig. 2). Furthermore, all final levels of adaptation differed significantly from 30° (0.35 s: *W* = −210.0, *P* < 0.001, 95% CI = (−5.35, −2.39); 1 s: *W* = −204.0, *P* < 0.001, 95% CI = (−6.87, −2.98); 2.5 s: *W* = −210.0, *P* < 0.001, 95% CI = (−8.09, −3.25)], with adaptation reaching 84.7%, 82.8%, and 82.0% of the optimum, respectively (Fig. 3).

In summary, our results support previous work suggesting that mental rotation contributes an interval to the planning time required to counter visuomotor rotations, which is proportional to rotation magnitude. Further, short periods of preparation interrupt planning, leading to greater residual errors. However, for the smallest rotation, we found no relationship between preparation time and error, suggesting that mental rotation was completed within the allowed time. But even in these circumstances, there were significant residual errors. In fact, in opposition to results reported by Langsdorf et al. (9), all groups displayed incomplete adaptation regardless of the preparation time provided.

### Restricting Motor Preparation Time Resulted in a Stronger Implicit Retention

Implicit retention (mean reach angle in the first 16 no-feedback trials, two cycles of reaches to each target location) was compared between groups in a two-way ANOVA (Rotation Magnitude × Preparation Time). Results from the ANOVA revealed a significant main effect of rotation magnitude [*F*(2,171) = 6.71, *P* = 0.002, ${\text{η}}_{\text{p}}^{2}$ = 0.07] and preparation time [*F*(2,171) = 18.33, *P* < 0.001, ${\text{η}}_{\text{p}}^{2}$ = 0.18] but no significant interaction [*F*(4,171) = 1.08, *P* = 0.37, ${\text{η}}_{\text{p}}^{2}$ = 0.03]. Multiple comparisons showed that participants in the restricted 0.35-s motor preparation time groups displayed greater retention than both the 1-s (*P* < 0.001) and 2.5-s (*P* < 0.001) groups, with no differences between the latter two (*P* > 0.99).

### Input Device and Handedness Had No Effect on Late Adaptation Levels

To determine whether the input device used (trackpad or mouse) or the handedness of the participant had any effect on our main outcome variable, reach angle, we ran an additional mixed-design ANOVA (Rotation Magnitude × Preparation Time) with factors for both input device and handedness (online groups only). The ANOVA revealed no significant main effect of input device [*F*(1,150) = 0.9, *P* = 0.35, ${\text{η}}_{\text{p}}^{2}$ = 0.006] or handedness [*F*(1,150) = 1.12, *P* = 0.29, ${\text{η}}_{\text{p}}^{2}$ = 0.007]. In fact, no interaction that included the term input device revealed significance [Rotation Magnitude × Input Device: *F*(2,150) = 0.36, *P* = 0.7, ${\text{η}}_{\text{p}}^{2}$ = 0.005; Preparation Time × Input Device: *F*(2,150) = 0.23, *P* = 0.79, ${\text{η}}_{\text{p}}^{2}$ = 0.003; Rotation Magnitude × Preparation Time × Input Device: *F*(4,150) = 0.3, *P* = 0.88, ${\text{η}}_{\text{p}}^{2}$ = 0.008; Supplemental Fig. S1, see https://doi.org/10.6084/m9.figshare.14797926). Similarly, all interaction effects with handedness were also nonsignificant [Rotation Magnitude × Handedness: *F*(2,150) = 1.55 *P* = 0.22, ${\text{η}}_{\text{p}}^{2}$ = 0.02; Preparation Time × Handedness: *F*(2,150) = 0.75, *P* = 0.48, ${\text{η}}_{\text{p}}^{2}$ = 0.01; Rotation Magnitude × Preparation Time × Handedness: *F*(2,150) = 0.12, *P* = 0.89, ${\text{η}}_{\text{p}}^{2}$ = 0.002]. These analyses corroborate our belief that neither the handedness of the participants nor the input devices they used confound our results.

### There Were No Differences in Task Performance between the Online and In-Laboratory Groups

**Figure 4.**
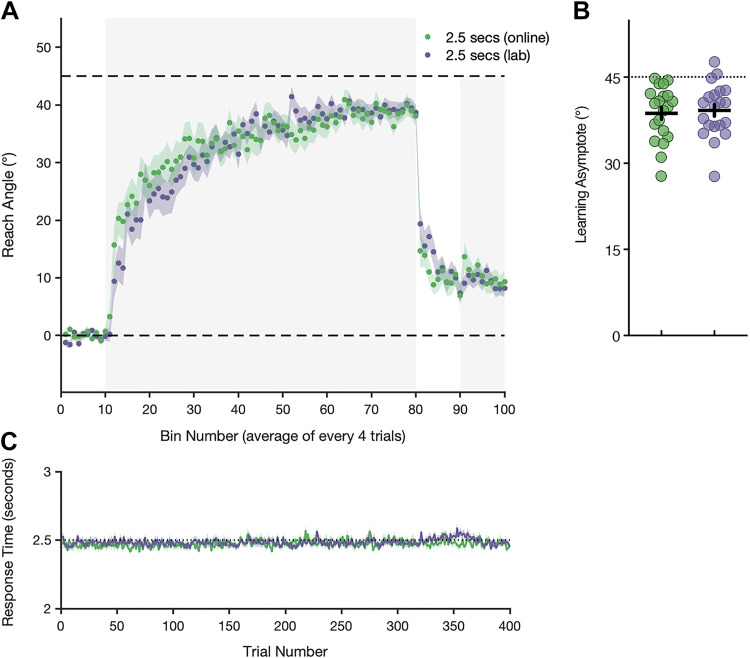
Mean reach angle, response time, and learning asymptote for the in-laboratory and comparative online experimental group. *A*: reach error (±standard error, shaded region) is averaged every four trials into during baseline, adaptation (shaded gray background), no feedback, and de-adaptation trials (shaded gray background) for each group. Zero degrees and the magnitude of imposed rotation are shown by horizontal dashed lines. *B*: the asymptotic level of adaptation for each participant in both the online and in-lab group. Filled circles represent the adaptation asymptote for each participant (defined as the average of the last 40 adaptation trials), with mean values and standard error depicted by horizontal and vertical lines, respectively. Dashed horizontal lines depict the imposed cursor rotation. *C*: mean response times (time between target presentation and movement initiation), ±standard error (shaded region) for each group. Preparation time was predetermined and tightly controlled at 2.5 s.

To confirm the validity of our results collected using online methodology, we collected data from an additional group of participants who performed the experiment in a laboratory with 2.5 s of preparation time and a 45° cursor rotation. Data from this additional in-laboratory experimental group were compared with the equivalent online group using unpaired *t* tests (two-tailed) and revealed no significant differences in baseline, asymptotic, or retention performance [*t*(38) = 0.89, *P* = 0.38; *t*(38) = 0.35, *P* = 0.73; *t*(38) = 1.78, *P* = 0.084, respectively]. Participants in this group compensated 87.2% for the rotation, and importantly, the final level of adaptation was also significantly different from 45° [*W* = −196.0, *P* < 0.001, 95% CI = (−8.35, −3.1)], replicating the effect found consistently across our online groups (Fig. 4). We also found no differences in variance between the laboratory and online groups in each of the experimental phases [*F* tests: *F*(19,19) = 1.9, *P* = 0.17; *F*(19,19) = 1.04, *P* = 0.93; *F*(19,19) = 1.31, *P* = 0.56].

## DISCUSSION

We aimed to test the assumption that increased motor preparation periods may allow for more complete adaptation during visuomotor rotation tasks. As such, we hypothesized that shorter preparation periods would be sufficient to fully counteract a 30° rotation, as a small rotation would require less (and therefore quicker) mental rotation before motor execution. We then predicted that this effect would scale with an increased cursor rotation, so that 45° and 60° would require greater mental rotation and thus more time to fully compensate. Indeed, we did find that restricting planning time for moderate and larger rotations resulted in impaired final adaptation levels and greater implicit retention compared with those groups with more preparation time. However, contrary to research from Langsdorf et al. (9), we found no evidence to suggest that extended motor preparation periods allow for the elimination of residual errors observed during late stages of adaptation, as all groups displayed final adaptation performance significantly lower than the imposed cursor rotation.

Notably, participants’ failure to fully compensate for visuomotor rotations under the conditions of the present study differs from the findings of Langsdorf et al. (9), who observed that participants were able to fully compensate for visuomotor rotations when provided with sufficient planning time. The authors suggested that the extended planning period allowed for time-consuming cognitive processes, such as mental rotation, to be completed before the go-signal and resulted in complete compensation. This theory, centered around a speed-accuracy trade-off, seems plausible given that reaction times increase in a linear fashion when subjects are asked to reach at increasing angles from a target—a relationship that mimics the behavior seen in mental rotation tasks (20, 21, 26). Accordingly, brain regions activated in both visuomotor adaptation and mental rotation tasks have also been shown to overlap (29), suggesting that mental rotation contributes to visuomotor adaptation. Recent evidence investigating preparatory activity in the motor cortex of nonhuman primates further suggests the necessity for motor planning time in visuomotor adaptation (30). In addition, Langsdorf et al. (9) found that delaying movement initiation resulted in an overcompensation of the imposed rotation, similar to the overshoot in reach estimation shown by Georgopoulos and Massey (21), suggesting comparable cognitive rotation strategies may have been in play.

Notwithstanding these studies, we found that, on average, participants’ learning plateaued before eliminating error, with asymptotic learning levels statistically different from the degree of imposed rotation for all rotational groups (30°, 45°, and 60°), regardless of the amount of preparation time enforced. These findings are consistent with previous reports which have suggested that sustained errors during visuomotor adaptation are a product of the implicit learning system, either suppressing alternative learning mechanisms that may be able to overcome persisting errors (7, 31) and/or modulating the system’s sensitivity to errors dependent on their size, variability, and history (32). It should be noted, however, that despite regression analysis suggesting the data had reached an asymptote, there is a possibility that with a longer adaptation session, or with multiple sessions, participants may have been able to reduce their error further and get closer to complete compensation. Yet, given the degree of error remaining at the end of these 280 trials, and the nonsignificant slopes of the regression analyses, it is unlikely that much greater adaptation would be achieved with additional trials. In support of this, estimated asymptotic levels of adaptation modeled from existing mean group data are in close correspondence with the values calculated in our original analysis and suggest all groups would asymptote below the degree of imposed rotation (Supplemental Fig. S2; see https://doi.org/10.6084/m9.figshare.17136857).

One factor that may explain the discrepancy between our results and those of Langsdorf et al. (9) is the level of experimental control. Our study was conducted online, with participants completing the task unsupervised at home, whereas Langsdorf et al. (9) conducted a laboratory-based study. The disparities associated with these environments may have contributed to small but significant differences in motivation, attention, and behavior. That said, data from our additional in-laboratory group are at odds with this reasoning. We found no difference in adaptive performance at any phase of the task between the in-laboratory and corresponding online group, with participants in both groups failing to achieve full compensation. This finding supports previous literature which suggests that kinematic data and results from online motor learning studies are analogous with those collected in a controlled laboratory environment (33) and that online tasks perform well in terms of accuracy and precision with regard to timing of visual stimuli and response capture (34). Furthermore, our data are typical of many previous laboratory-based visuomotor adaptation studies, with comparable levels of final performance (5, 7, 8, 35–37). With all of these results in mind, it seems improbable that the failure to eliminate residual errors [and replicate results from the study by Langsdorf et al. (9)] was a consequence of online data collection methods.

A further notable difference between the two studies is the apparatus used to complete the task. In the present study, participants either used a trackpad or a mouse to direct the cursor toward targets. We expect these movements demanded rotation of the wrist with flexion/extension of the index finger (trackpad) and small movements about the elbow/shoulder joint (mouse). The sensory and biomechanical control of these movements likely differ from that noted in the study by Langsdorf et al. (9), where a digitizing tablet was used to make small point-to-point reaching movements while holding a stylus and may have contributed to some discrepancy in results between the two studies (38, 39). However, as we found no differences between the mouse and trackpad conditions, which themselves entail very different biomechanical demands, we believe our results generalize across methodologies. Moreover, in contrast to Langsdorf et al. (9), we did not ask participants to cover or block vision of their upper-limb during the task. As a result, there may have been some differences in the explicit awareness of the cursor rotation and thus performance during adaptation (40).

An additional difference between the two studies, which may have had some bearing on the results, is the nature of response cueing used. We used a visual countdown sequence that enabled participants to accurately synchronize their movements with the go-signal and ensured a tight coupling between response times and the preparation time groupings. In contrast, Langsdorf et al. (9) displayed the target and instructed participants to wait until they heard a tone before responding. This difference in protocol resulted in response times closer to 3 s. One may argue that more than 2.5 s are required to fully prepare for a 45° rotated reach. This is unlikely however, as we show that even a 30° angle was not fully compensated, and there was no evidence for a relationship with the preparation interval for those groups. Previous research has also shown that mental rotation and aiming toward angles up to 90° can be achieved in ∼1 s (20). There is perhaps an argument to suggest that attending to the countdown sequence may have interfered with mental rotation and other cognitive planning processes, by, for instance, vying for attentional resources and thus contributing to a consistent reach undershoot (41, 42). Nevertheless, prior studies using auditory response cueing have not cited attention to the timed-response sequence as a potential confound (20, 27, 43).

Despite participants failing to eliminate residual errors during late adaptation, our data do include some hallmarks of a speed-accuracy trade-off. We found that motor adaptation was impaired in the 45° and 60° rotational groups, during both early and later stages, when planning time was restricted to 0.35 s. This result is in line with previous studies that reported reduced error compensation when preparation times are limited (4, 32, 43, 44) and may reflect the suppression of explicit reaiming/cognitive strategies. In addition, impaired adaptation associated with restricted preparation times was coupled with an increased retention during no-feedback trials, which is consistent with the idea that learning involved more implicit, procedural processes (4, 27). The greatest retention was seen in the 0.35 s/60° group, which implies that adaptation in this group may have been weighted more so toward implicit processes—also highlighted by the larger residual errors at the end of learning. This suggestion is reinforced by the equal retention seen for the 2.5-s condition, across all three rotation magnitudes. In this case, the residual errors are approximately equal, because the preparation time exceeded that required for mental rotation. However, these assumptions should be treated with some caution after recent commentary on how the dichotomy of implicit and explicit components of motor adaptation is inferred (45).

Although not statistically evident, a speed-accuracy trade-off may also be apparent in the 60° rotational group (Fig. 3). On average, there was a linear increase in learning asymptote as preparation time increased, which may reflect how mental rotation of the intended movement angle increases the accuracy of reaches (9, 20). The addition of further adaptation trials might exaggerate these asymptotic differences until statistically significant, yet data from our other rotational groups, modeling, and previous literature would suggest that errors would still persist. To achieve full compensation, it is possible that explicit information about the nature of rotation and how to counter it ought to be provided (9, 14, 32, 46, 47), which will inherently be coupled with increased reaction/plan times.

In summary, our data suggest that extending motor preparation and planning periods alone is insufficient to eliminate residual errors during visuomotor adaptation, irrespective of the size of imposed cursor rotation. Although increased preparation time may help to improve error reduction at larger rotation magnitudes, our results suggest there remains a limit at which learning saturates at asymptote, perhaps only overcome with priming of explicit strategies, further instruction, or changes to experimental variables. We also provide further support of the use of online data collection methods in the study of motor control and learning. Understanding why the central nervous system fails to fully adapt movements in response to environmental changes may be key when aiming to optimize rehabilitation protocols following brain injury or disease.

## SUPPLEMENTAL DATA

10.6084/m9.figshare.14797926 Supplemental Fig. S1: https://doi.org/10.6084/m9.figshare.14797926.

10.6084/m9.figshare.17136857 Supplemental Fig. S2: https://doi.org/10.6084/m9.figshare.17136857.

## GRANTS

This work was funded by the MRC-Versus Arthritis Centre for Musculoskeletal Ageing Research (CMAR). R.C.M. was also partly funded by the Royal Society, Leverhulme and Wellcome Trust.

## DISCLOSURES

No conflicts of interest, financial or otherwise, are declared by the authors.

## AUTHOR CONTRIBUTIONS

M.W., J.-S.B., R.C.M., and N.J. conceived and designed research; M.W. performed experiments; M.W., J.-SB., R.C.M., and N.J. analyzed data; M.W., J.-S.B., R.C.M., and N.J. interpreted results of experiments; M.W. prepared figures; M.W. drafted manuscript; M.W., J.-S.B., R.C.M., and N.J. edited and revised manuscript; M.W., J.-S.B., R.C.M., and N.J. approved final version of manuscript.
